# Reversal of neuronal tau pathology via adiponectin receptor activation

**DOI:** 10.1038/s42003-024-07391-z

**Published:** 2025-01-04

**Authors:** Eric R. McGregor, Danny J. Lasky, Olivia J. Rippentrop, Josef P. Clark, Samantha Wright, Mathew V. Jones, Rozalyn M. Anderson

**Affiliations:** 1 Division of Geriatrics, Department of Medicine, SMPH, University of Wisconsin-Madison, Madison, WI USA; 2 Department of Nutritional Sciences, University of Wisconsin-Madison, Madison, WI USA; 3 Department of Neuroscience, University of Wisconsin–Madison, Madison, WI USA; 4 GRECC William S. Middleton Memorial Veterans Hospital, Madison, WI USA

**Keywords:** Energy metabolism, Excitability, Autophagy, Mechanisms of disease, Transcriptomics

## Abstract

Changes in brain mitochondrial metabolism are coincident with functional decline; however, direct links between the two have not been established. Here, we show that mitochondrial targeting via the adiponectin receptor activator AdipoRon (AR) clears neurofibrillary tangles (NFTs) and rescues neuronal tauopathy-associated defects. AR reduced levels of phospho-tau and lowered NFT burden by a mechanism involving the energy-sensing kinase AMPK and the growth-sensing kinase GSK3b. The transcriptional response to AR included broad metabolic and functional pathways. Induction of lysosomal pathways involved activation of LC3 and p62, and restoration of neuronal outgrowth required the stress-responsive kinase JNK. Negative consequences of NFTs on mitochondrial activity, ATP production, and lipid stores were corrected. Defects in electrophysiological measures (e.g., resting potential, resistance, spiking profiles) were also corrected. These findings reveal a network linking mitochondrial function, cellular maintenance processes, and electrical aspects of neuronal function that can be targeted via adiponectin receptor activation.

## Abstract (web summary)

Activation of adiponectin receptor signaling in tauopathy-prone primary neurons clears tangles through activation of lysosomes and rescues mitochondrial energetics, lipid storage disorder, neurite outgrowth, and electrophysiological defects

## Introduction

The specific mechanisms for cognitive decline and dementia associated with Alzheimer’s disease (AD) remain unclear; however, the loss in cognitive performance is more strongly correlated with neurofibrillary tangles (NFT) than with amyloid-beta (Aβ) plaques^1,2^. NFTs are composed of aberrant hyper-phosphorylated tau aggregates, a microtubule-associated protein encoded by the MAPT gene. Ordinarily, tau plays a key role in neuronal structure and trafficking of cargo along axons^3,4^. NFTs also accumulate in other neurodegenerative diseases, such as frontotemporal dementia, Parkinson’s disease, and chronic traumatic encephalopathy, so mechanisms underlying NFT formation and interventions for NFT clearance would have broad clinical significance.

Disruptions in metabolic pathways are coincident with the development of AD pathology, whether spontaneous or engineered^5,6^. Adverse changes in mitochondrial energetics have been linked to tauopathy^7–10^, and disruption in mitochondrial morphology, proteomics, interactome, and mitophagy have been reported in various tauopathy mouse models^11–14^. Mitochondrial dysfunction is a hallmark of the aging process and may play a causal role in the increase in risk for neurodegenerative disease onset as a function of age. The idea that preserving mitochondrial function might be effective in blunting pathology and improving neural function is gaining traction^15,16^, although precisely how this might be accomplished remains to be shown.

One potential therapeutic target for activating mitochondrial metabolism is adiponectin receptor agonism. Adiponectin is a highly abundant 30 kDa peptide hormone secreted by adipose tissue that regulates metabolism in its target tissues, increasing mitochondrial activity and fatty acid oxidation, and is associated with systemic insulin sensitivity^17,18^. Adiponectin counters metabolic defects linked to obesity^19^, is linked to preserved healthspan in normative aging^20^, and is upregulated by the longevity-promoting intervention of caloric restriction^21,22^. Adiponectin signaling occurs through adiponectin receptors 1 and 2 (AdipoR1/2), with a poorly understood contribution from the T cadherin receptor^23^. AdipoRs are expressed in the brain^24^, and recent studies suggest that neuronal AdipoR activation may be neuroprotective in the context of diabetes and stroke^25,26^. AdipoRon (AR) is a nonselective adiponectin receptor agonist that binds to both AdipoR1/2^27^ and has been shown to promote aggregate clearance in models of AD^28,29^. The present study aimed to investigate the interactions between disease pathology, neuronal function, and neuronal metabolism.

## Results

### AdipoRon clears phosphorylated Tau and NFT via AMPK and GSK3b

**Fig. 1:**
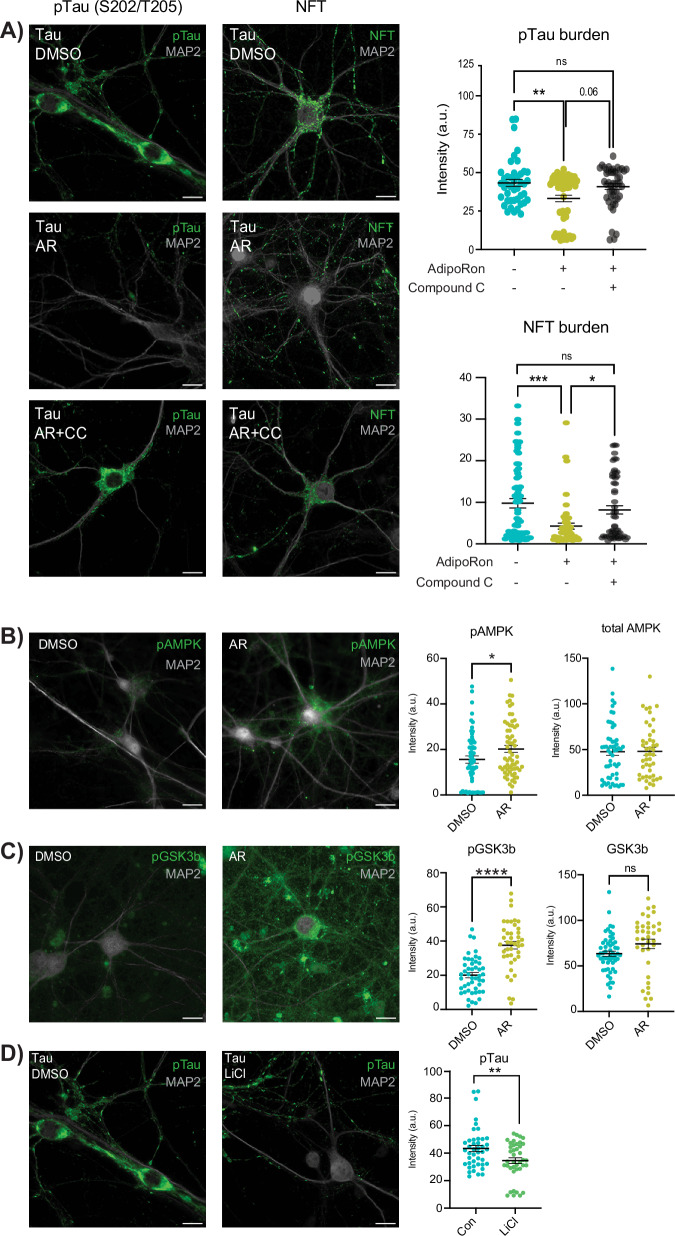
AdipoRon clears phosphorylated tau and NFT via AMPK and GSK3b. **A** Immunofluorescence of phosphorylated Tau (pTau) at S202/T205 and NFT (MC-1 antibody) in Tau neurons following 24-h treatment with 10 μM AdipoRon ± 8 μM AMPK inhibitor Compound C (pTau: *n* = 6 mice, *n* = 43–58 neurons per treatment; NFT: *n* = 8 mice, *n* = 53–79 neurons per treatment), quantification of immunofluorescence images above; one-way ANOVA with Tukey’s multiple comparison’s test, scale bar 15 μm. **B** Immunofluorescence detection and quantification of phosphorylated AMPK and total AMPK in individual Tau neurons following 10-min treatment with 10 μM AdipoRon (pAMPK: *n* = 5 mice, *n* = 57–63 neurons per treatment; total AMPK: *n* = 3 mice, *n* = 44–56 neurons per treatment); Student’s *t*-test with Welch’s correction. **C** Immunofluorescence detection and quantification of phosphorylated GSK3b and total GSK3b in Tau neurons following 10-min treatment with 10 μM AdipoRon (pGSK3b: *n* = 5 mice, *n* = 42–47 neurons per treatment; total GSK3b: *n* = 5 mice, *n* = 37–55 neurons per treatment), Student’s *t*-test with Welch’s correction. **D** Immunofluorescence detection of pTau after 24-h treatment with 15 mM LiCl (*n* = 6 mice; *n* = 40–43 neurons per treatment). Data shown as mean ± SEM *p < 0.05, **p < 0.01, ***p < 0.001, ****p < 0.0001.

Primary neurons carrying prion promoter-driven hTauP301S cDNA accumulate phosphorylated Tau and form neurofibrillary tangles, a process that is further augmented by seeding with pre-formed Tau fibrils (PFF). Isolated primary neurons from P0 neonate hTau P301S mouse pups (Fig. S1) were seeded with PFF^30^ on day seven and cultured for an additional 18–22 days to induce hyperphosphorylated Tau neurofibrillary tangles (NFT) (Fig. 1A, upper panels). In brains from tauopathy mice, modest differences in AdipoR expression have been reported, but the impact is age, strain, and brain region-specific (Fig. S1). The abundance of transcripts of AdipoR1, but not AdipoR2, appeared numerically lower in Tau neurons compared to WT, although this did not reach statistical significance (Fig. S1). With AR treatment, AdipoR1 expression in tau neurons was significantly higher than DMSO-treated controls and restored to WT levels (Fig. S1). Treatment with AR (10 μM) for 24 h significantly reduced levels of Tau phosphorylated at S202/T205 as detected by immunofluorescence (Fig.1A, left panels) or by western blot and dot blot (Fig. S1). Furthermore, lower levels of total NFT were detected using a tangle-specific antibody (Fig. 1A, right panels)^31^. The best-characterized mediator of adiponectin signaling is AMPK, which is thought to become activated downstream of AdipoR1/2 ligand binding^18^, although most evidence comes from non-neuronal cell types. Treatment with AMPK inhibitor Compound C (8 μM) blunted the clearance of hyperphosphorylated Tau and NFT in seeded neurons, indicating that reversal of Tau pathology via AR is AMPK-dependent (Fig. 1A, lower panels). An alternate inhibitor of AMPK, BAY3827 (1 μM), yielded the same outcome (Fig. S1). These data suggest a role for AMPK but do not reveal if it is specifically activated in response to AR in primary neurons. Intracellular signaling by phosphorylation tends to be rapid and transient, prompting an investigation over a shorter timeframe. Seeded primary neurons were treated for 10 min with 10 μM AR or DMSO control, at which time significantly greater activating phosphorylation at S172 was detected (Fig. 1B). Total AMPK was not significantly different in either treated or control neurons over this same time frame (Figs. 1B and S2).

AMPK is not known to be directly involved in Tau phosphorylation; however, a strong candidate is the established Tau kinase GSK3b (glycogen synthase kinase 3 beta). NFT accumulation has been shown to lead to increased acetylation of GSK3b, modulating its activity and creating a feedforward cycle^32^. Prior independent studies have suggested that it might also play a role in adiponectin signaling^33,34^, and GSK3b is known to be directly inhibited by AMPK^35,36^. Tau neurons treated with AR for 10 min contained significantly greater inhibitory phosphorylation of GSK3b at the Ser9 residue (Fig. 1C), while total GSK3b remained unchanged (Fig. S2). To confirm prior reports that GSK3b inhibition directly leads to a lower level of phosphorylated Tau, Tau neurons were treated with 15 mM lithium chloride (LiCl), a well-documented GSK3b inhibitor. Within 24 h of LiCl treatment, phosphorylated Tau (S202/T205) was significantly lower, indicating that GSK3b inhibition is sufficient to reverse Tau modification in primary neurons (Fig. 1D). These data place AMPK and GSK3b downstream of AR in clearing the aggregate-prone Tau from pre-seeded primary neurons and show that NFT formation is dynamic and reversible via activation of adiponectin signaling.

### Neuronal transcriptional response to AdipoRon extends beyond metabolic regulation

Initially, AR was developed to correct defects in metabolism linked to obesity^27^; however, changes in metabolism can have broad cellular effects^37^. To gain insight into the impact of NFT burden and the response to AR, transcriptional profiling was conducted via RNAseq. RNA was extracted from WT and P301S neurons 18 days post-PFF seeding and following 24-h treatment with 10 μM AR. Almost 1.6 billion sequencing reads were detected (~78 million reads per sample), trimmed, and aligned to the GRCm39 mouse genome, yielding ~42,000 transcripts from 20,000 unique genes. Differential expression analysis was conducted among the four groups (WT and Tau, with or without AR). Consistent with prior reports, the transcriptional response to the presence of NFTs was negligible^38,39^. Only one gene, Gbp2b (guanylate binding protein 2b), was differentially expressed between Tau and WT neurons and increased 6-fold. Gbp3b is a regulator of inflammatory responses previously associated with neuronal death in response to traumatic injury^40^.

In contrast, transcripts from 138 genes were differentially expressed between the treatment groups (AR vs. control) for Tau neurons (FDR < 0.05) (Fig. S3, Table S1). A greater number of significantly differentially expressed genes were detected in AR-treated WT neurons, 656 genes, including 137 of those detected in the Tau neurons for which the directionality of change was conserved (Fig. S3). Top AR-responsive genes were dispersed among cellular functions and included Nptx2 (synaptic communication), Kcnv1 (potassium channel), Sost (Wnt Inhibitor), Ucn (Dopaminergic neuron differentiation), Lyve1 (Membrane glycoprotein), Adcyap1 (Stress response), Pbp2 (MAPK cascade antagonist), Pdcd1 (Immune), and Chrnb3 (cholinergic receptor) (Fig. S3; Table S1). These data show that the impact of AR extends beyond metabolic modulation and suggest that broader aspects of neuronal function were harnessed downstream of adiponectin receptor activation.

**Fig. 2:**
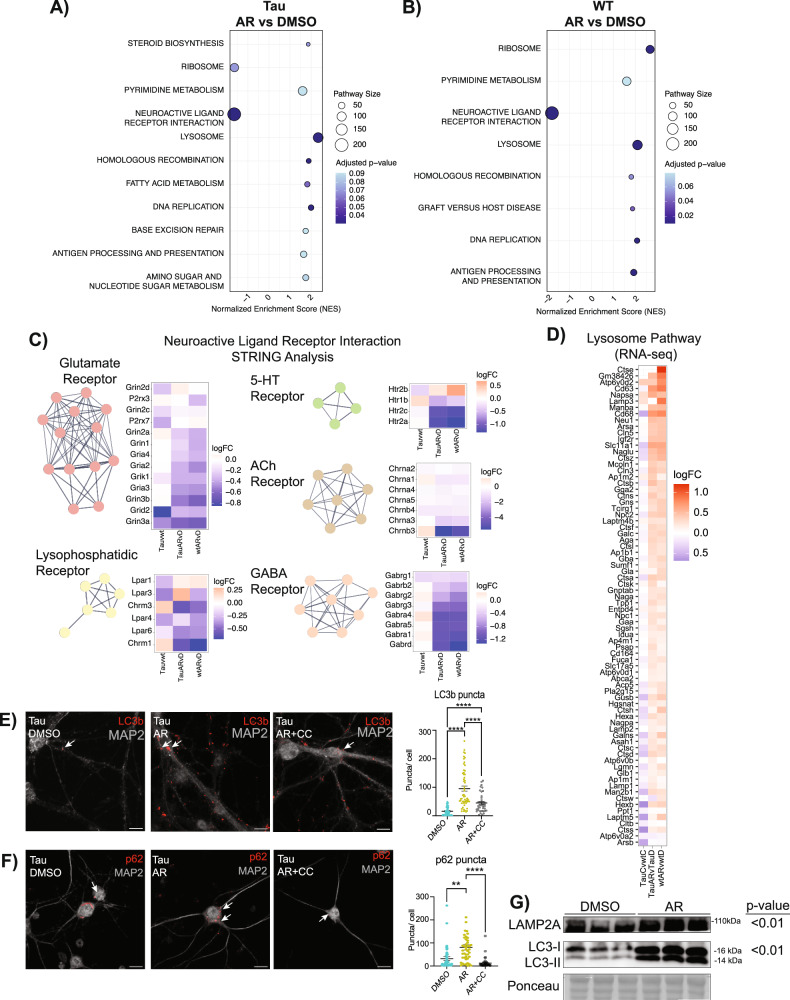
Neuronal transcriptional response to AdipoRon extends beyond metabolic regulation. **A** Pathway enrichment was determined via GSEA of tau neuron AR-responsive KEGG pathways using RNA-seq data (*n* = 5–6 pups per genotype per treatment). **B** As above for AR-responsive KEGG pathways in WT neurons. **C** STRING analysis and heatmap displaying log_2_FC of Neuroactive Ligand Receptor Interaction Pathway genes sub-grouped via Markov Cluster Algorithm (clusters ≥ 4 genes shown) in Tau neurons in response to AR. **D** Heatmap of lysosome pathway detected by GSEA in AR-treated Tau and WT neurons compared to untreated WT. **E**, **F** Immunofluorescent detection and quantification of autophagosome markers LC3b (*n* = 4–6 mice, *n* = 33–56 neurons per treatment) and p62 (*n* = 3 mice, *n* = 29–43 neurons per treatment) in Tau neurons after 24-h treatment with 10 μM AdipoRon ± 8 μM Compound C. **G** Western blot of LAMP2A and LC3 with representative ponceau in neurons after 24-h treatment with 10 μM AdipoRon (LAMP2A: *n* = 6 mice per treatment; LC3: *n* = 9 mice per treatment). Significance determined by one-way ANOVA with Tukey’s multiple comparison’s test or Student’s *t* test. Data shown as mean ± SEM *p < 0.05, **p < 0.01, ***p < 0.001, ****p < 0.0001.

Kyoto Encyclopedia of Genes and Genomes (KEGG) pathway analysis via gene-set enrichment analysis (GSEA) identified AR-responsive pathways in Tau neurons (Fig. 2A) and in WT neurons (Fig. 2B). Among those shared pathways positively enriched were proteostasis, DNA maintenance and repair, and metabolism. Ribosomal pathways were enriched in both WT and Tau neurons but in opposite directions, perhaps reflecting change in ribosomal composition rather than in ribosomal abundance^41^. Homologous recombination and DNA replication pathways were AR-responsive in both genotypes, with base excision repair also detected in the AR-treated Tau neurons. The biology behind this is unclear, and a connection between genome maintenance and adiponectin has not previously been identified.

The most highly enriched pathway for both genotypes was neuroactive ligand-receptor interactions. This pathway is composed of the families of receptors involved in synaptic communication as well as many of their ligands. STRING analysis of genes identified within this pathway detected 5 clusters (Fig. 2C). The glutamate receptor cluster contained genes encoding six NMDA, three AMPA, one kainate, and one delta subunit. These subunits are associated with ionotropic glutamatergic excitability, suggesting a lowering of excitatory postsynaptic responses with AR, which would protect against excitotoxicity^42^. The GABA_A_ receptor transports Cl^-^ ions, typically inhibiting spike firing by hyperpolarization or shunting. Therefore, a decrease in expression would increase the probability of postsynaptic firing. Nicotinic ACh receptors are also ionotropic, allowing cations to flow and elicit rapid depolarizations. Serotonin (5-hydroxytryptamine, 5-HT) receptors are associated with both metabotropic and ionotropic function and fine-tuning cortical neuron behavior. Serotonin receptor activation has been linked to both amyloid-beta secretion and tau phosphorylation^43,44^. The lysophosphatidic acid receptor cluster included lysophosphatidic acid receptors (Lpar) and metabotropic acetylcholine receptors. Lpar activation is linked to in vitro neurite retraction, neuronal network formation, and forebrain development^45^, while metabotropic ACh receptor activation leads to the hydrolysis of phosphatidylinositol 4,5 bisphosphate^46^. Together, these transcriptional data suggest that AR impinges on neuronal communication processes, although it is not clear if this is coincident with or a consequence of clearance of the NFT.

### AdipoRon activates the lysosomal pathway via AMPK

Autophagy is a conserved cell process for the degradation of aggregates and dysfunctional organelles. Autophagosome-lysosome fusion allows degradative enzymes of the lysosome to access sequestered protein aggregates and damaged organelles^47^. Autophagy has been proposed as a potential target for alleviating both Aβ plaques and NFTs^48,49^, and lysosomal dysfunction has been implicated in AD progression^50^. Lysosome was one of the most significantly positively enriched pathways in AR-treated Tau and WT neurons (Fig. 2D), and there is a hint that this pathway is impacted by Tau, although significance in that case was not reached. AMPK is an established regulator of autophagy^51^, acting through ULK and mTOR to stimulate autophagosome maturation. Activated AMPK increases the formation of autophagosomes that can be visualized by detecting LC3b (microtubule-associated protein 1a/1b light chain 3b) associated puncta^52^. Exposure of Tau neurons to AR (10 μM) for 24 h (matching the RNAseq data) significantly increased the number of LC3b puncta (Fig. 2E) in an AMPK-dependent manner, with significantly fewer puncta detected in the presence of Compound C (8 μM). Treatment with the alternate inhibitor of AMPK BAY3827 (1 μM) yielded the same outcome (Fig. S3). The chaperone protein p62 shuttles proteins to the autophagosome for subsequent degradation, and it has been previously linked to the anti-inflammatory effects of adiponectin^53^. A greater number of p62-positive puncta were detected following 24 h of AR treatment (Fig. 2F), again in an AMPK-dependent manner. Treatment with AR also led to the increase of LAMP2A, the LAMP2 variant essential for chaperone-mediated autophagy (Fig. 2G). To address the possibility that autophagy was stalled rather than activated, LC3 conversion was investigated by immunoblotting. AR treatment led to an increase in LC3-II expression and an increase in the ratio of LC3-II/ LC3-I, suggesting that AR activates autophagy (Fig. 2G, Fig. S3). These data show that autophagosome induction occurs after AR treatment and suggest that this AMPK-dependent process may contribute to NFT clearance.

### AdipoRon rescues mitochondrial deficits caused by tauopathy

**Fig. 3:**
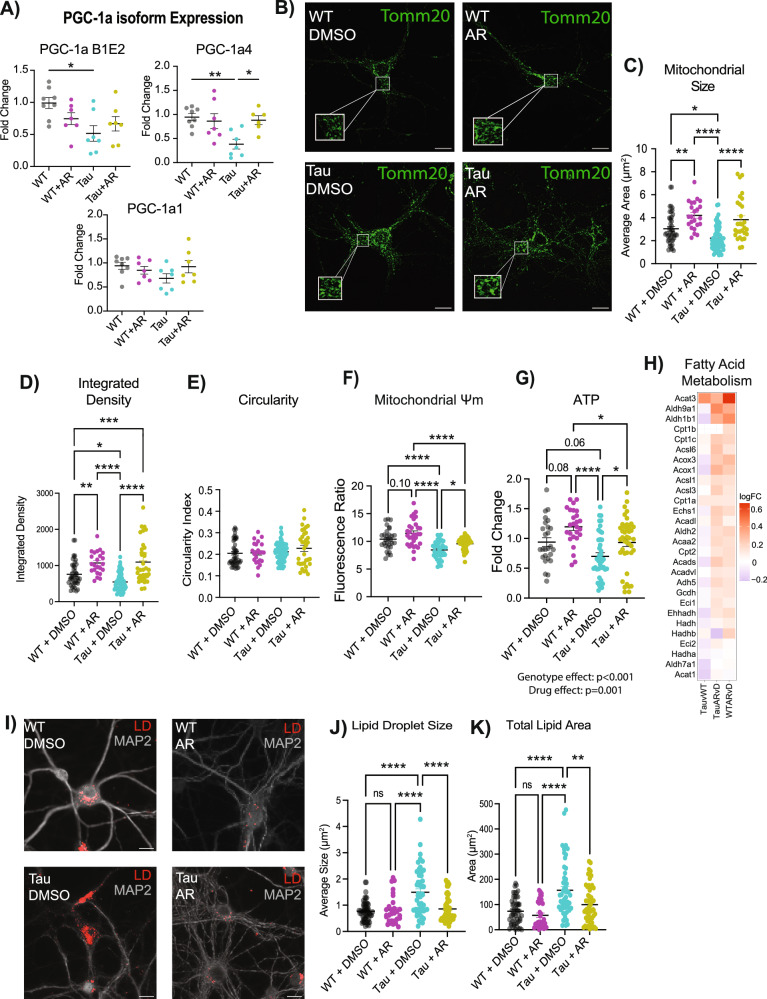
AdipoRon rescues mitochondrial deficits caused by tauopathy. **A** mRNA abundance of Ppargc-1a isoforms was determined by RT-qPCR (*n* = 6–8 mice per treatment per genotype). **B** Representative images of mitochondria detected with TOMM20 antibody. **C** Quantification of mitochondrial size, (**D**) Integrated density, and (**E**) circularity (*n* = 6 mice per genotype, *n* = 23–59 cells neurons per treatment per genotype. **F** Mitochondrial membrane potential (JC-1) 2 h after 10 µM AR treatment (*n* = 5–6 mice per genotype; *n* = 30–43 observations per genotype per treatment). **G** Relative ATP concentrations after 12 h of AdipoRon treatment (*n* = 8–12 mice per genotype, *n* = 26–41 observations per treatment per genotype). **H** Heatmap of log_2_FC of fatty acid metabolism GSEA pathway genes in AR-treated WT and Tau neurons with Tau compared to WT included for reference. **I** Representative images of LipidTox dye (scale bar 15 µM). **J**, **K** Quantification of lipid droplet size and total area (*n* = 5 mice per genotype, *n* = 30–55 neurons per treatment per genotype). Significance determined by multiple Student’s *t*-test or two-way ANOVA with Tukey’s multiple comparisons test. Data shown as mean ± SEM *p < 0.05, **p < 0.01, ***p < 0.001, ****p < 0.0001.

It has been established that metabolic dysfunction occurs in the brain during aging and Alzheimer’s disease, with mitochondrial dysfunction thought to be a critical consequence of disease pathology^54,55^. PGC-1a is a transcriptional coactivator and master regulator of mitochondrial function that has been indirectly linked to broader cellular processes in diverse cell types, including redox metabolism, cell cycle and cell size determination, arborization, and hypertrophy^37,56,57^. Three promotor regions in the Ppargc-1a gene are active in neurons, including one that is neuron-specific^58^, yielding three transcript variants of PGC-1a. Expression from two of the three promoters was significantly lower in Tau neurons compared to the WT (Fig. 3A). Our previous studies in murine fibroblasts had revealed a temporal aspect in response to AR treatment, where signaling to AMPK was transient and early, and increases in PGC-1a and its target genes were detected about an hour later^59^. A 90-min AR treatment (10 μM) had an isoform-specific effect on PGC-1a, restoring PGC-1a4 expression in the Tau neurons to WT levels. PGC-1a B1E2 expression was also increased in Tau neurons following AR treatment and was no longer significantly lower than WT levels (Fig. 3A). There were no significant effects of 10 μM AR on PGC-1a expression in WT neurons.

Mitochondrial morphology is dynamic and changes in response to metabolic status, with more reticular organization associated with a more oxidative setting^60^. Morphology was assessed by immunofluorescent detection of TOMM20 (translocase of outer mitochondrial membrane 20) (Fig. 3B). NFT burden was associated with significantly lower mitochondrial size (Fig. 3C) and integrated density (Fig. 3D), while the circularity index of the mitochondria was not different (Fig. 3E). AR treatment for 24 h (10 μM) restored mitochondrial size and integrated density to that of WT neurons. AR also increased mitochondrial size and integrated density in WT neurons, indicating that the drug may have utility in the absence of AD pathology. Next, the functional consequence of AR treatment on neuronal mitochondrial activity was assessed. Mitochondrial functional measures in response to AR treatment are observable at different times of AR exposure^59^. As such, the measures we observed (PGC-1a expression, mitochondrial morphology, membrane potential, and ATP levels) were taken at distinct times. Mitochondrial membrane potential is created by electron transport chain activity and drives the ability to derive ATP from oxidative metabolism. Proton motive force quantified by JC-1 assay was lower in Tau neurons (Fig. 3F), aligning with the depression in PGC-1a expression. Treatment with AR (10 μM) rescued this phenotype within 2 h and increased mitochondrial membrane potential in WT neurons. ATP availability was lower in Tau neurons compared to the WT, and AR treatment resulted in significantly greater ATP availability both in Tau neurons and in WT neurons (Fig. 3G).

Lipid accumulation is known to be a consequence of inefficient mitochondrial metabolism^29^, and lipid metabolic dysregulation has been linked to neurodegenerative disease progression^61^. AR treatment was associated with a positive enrichment of fatty acid metabolism as detected by RNASeq (Fig. 3H). These data suggested that lipid usage and lipid storage could be altered by AR treatment. Lipid droplets (LD) were detected using the neutral-lipid stain HCS LipidTox, revealing differences in lipid stores between WT and Tau neurons (Fig. 3I, Fig. S4). Quantitative analysis showed that LD size was significantly greater in neurons with Tau pathology (Fig.3J) and that the total area of lipid stain was significantly greater (Fig. 3K). AR treatment lowered LD size and total lipid area, restoring each to WT levels. Together, these data show diminished metabolic capacity and lipid accumulation in neurons with NFT, both of which can be rescued by AR-directed stimulation of the adiponectin pathway.

### AdipoRon restores dendritic complexity through the activation of JNK

**Fig. 4:**
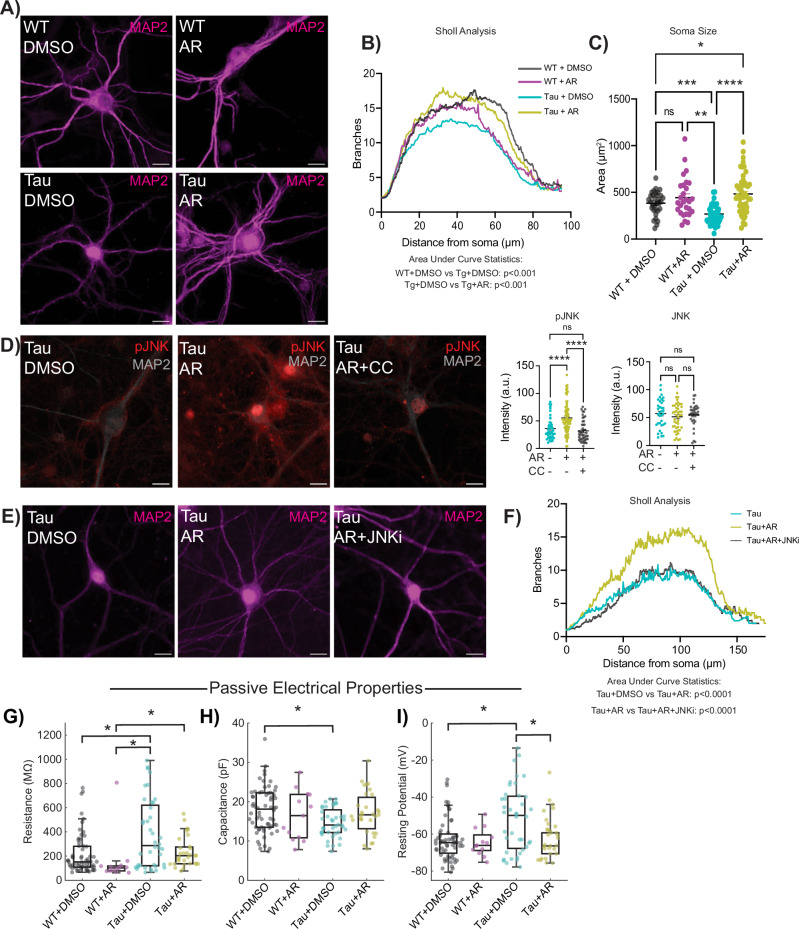
AdipoRon rescues loss of dendritic complexity through activation of JNK. **A** Representative immunodetection images of microtubule-associated protein 2 (MAP2) in WT and Tau neurons treated with 10 µM AR or DMSO. **B**, **C** Sholl analysis of dendritic branch number as a function of distance from soma and soma size (*n* = 4–5 mice per genotype, *n* = 28–51 neurons per treatment per genotype), two-way ANOVA with Sidak’s multiple comparisons test. **D** Representative immunodetection images and quantification of phosphorylated JNK (pJNK) (*n* = 4–6 mice per, *n* = 45–78 neurons per treatment) and total JNK (*n* = 3 mice per treatment, *n* = 34–45 neurons per treatment) following 10-min AR treatment (10 µM) ± 8 μM AMPK inhibitor Compound C, and (**E**) representative images of immunodetection of MAP2 in Tau neurons treated with 10 µM AR ± 10 µM JNK inhibitor SP600125. One-way ANOVA with Tukey’s multiple comparisons test. **F** Quantification of dendritic branch number and distance from soma determined by Sholl analysis (*n* = 3 mice per treatment; *n* = 47–51 neurons per treatment). Brown–Forsythe and Welch one-way ANOVA. Electrophysiological parameters of passive intrinsic excitability following 24-h 10 µM AR treatment including **G** resistance, (**H**) capacitance, and (**I**) resting potential. WT: 64 neurons, WT + AR: 13 neurons, Tg: 40 neurons, Tg + AR: 31 neurons. Kruskal–Wallis and post-hoc Dunn’s tests. Data shown as mean ± SEM, or as median ± IQR *p < 0.05, **p < 0.01, ***p < 0.001, ****p < 0.0001.

NFT aggregation is associated with a loss of dendritic spine density and dysfunction in microtubule interactions^62,63^, with implications for neuronal structural integrity. Dendritic arborization can be quantified via immunolabeling for microtubule-associated protein 2 (MAP2), a neuron-specific microtubule protein (Fig. 4A). MAP2 was excluded from the nucleus of WT neurons but not Tau neurons. The biological relevance of this difference in subcellular distribution is unclear; however, a similar phenotype has been observed in the brains of Parkinson’s disease patients^64^. Branch detection and scale estimation via Sholl analysis revealed significantly fewer dendritic branches in Tau neurons than in WT, suggesting a loss of dendritic complexity with NFT (Fig. 4B). AR treatment (10 μM, 24 h) rescued the NFT-associated compromise in dendrite outgrowth, and area under the curve analysis showed significant differences between Tau and WT neurons and between Tau neurons with or without AR. Although the shape of the curve was not identical for WT with or without AR, the change in the distribution of the data was not significant. The soma area was significantly smaller in Tau neurons than in WT, and AR restored soma size to WT values (Fig. 4C).

The stress-associated kinase JNK (c-Jun N-terminal Kinase) phosphorylates and stabilizes several microtubule-associated proteins, including MAP2 and MAP1B^65^, and is required for axonal growth^66^, maintenance of microtubes^65^, and outgrowth of neurons^67^. Adiponectin-induced activation of JNK has been previously reported^68^, but not in neurons, and the mechanism by which this occurs has yet to be established. AR treatment increased activating phosphorylation (T183/Y185) of JNK in Tau neurons (Fig. 4D) without impacting overall JNK levels (Fig. S5). Simultaneous treatment with AMPK inhibitor Compound C (8 μM) blocked the increase in JNK phosphorylation, placing the phosphorylation and activation of JNK in response to AR downstream of AMPK. Exposure of Tau neurons to JNK inhibitor SP600125 (10 μM) for 24 h blocked the neurite outgrowth induced by AR (Fig. 4E). Quantitation of the area under the curve for the distribution of number of branches as a function of distance from soma confirmed that JNK is required for the AR-induced dendritic arborization phenotype (Fig. 4F). These data show that JNK is activated by adiponectin receptor pathway in an AMPK dependent manner and is responsible for the AR-driven increase in dendritic complexity.

### Altered passive electrical properties in Tau neurons are restored by AdipoRon

Differences in soma size and dendritic complexity have functional consequences for the intrinsic electrical properties of neurons^69^. Using whole-cell patch clamp recording, we studied both passive and active electrical properties. Passive properties include membrane resistance, capacitance, and resting potential, whereas active properties include mechanisms underlying action potentials (see below). Membrane resistance was significantly elevated in Tau neurons compared to WT, but following 24-h treatment of 10 μM AR, membrane resistance was equivalent to WT neurons (Fig. 4G). Capacitance was reduced in Tau neurons compared to WT (Fig. 4H), aligning with the reduction in cell body size and dendritic branching, and was also restored to WT by AR. The resting potential is influenced by resting conductances and transmembrane ionic gradients. Tau neurons were relatively depolarized compared to WT (Fig. 4I), but AR treatment restored Tau neuron resting potential to WT values (Fig. 4I). Collectively, these results suggest that Tau neurons have altered passive electrical properties and that these impairments are restored by treatment with AR. The impact of NFT on electrical properties in neurons raised the possibility that this phenotype might extend to other aspects of AD pathology. Amyloid beta accumulates in the brain and vasculature with AD^70^, and this pathology can be induced in transgenic mice that express a chimeric mouse/human amyloid precursor protein carrying the 695 Swedish mutation (APP^SWE695^) and a mutant human presenilin 1 (PS1-dE9). There were no statistical differences in membrane resistance, capacitance, or resting potential between APP/PS1 and WT primary neurons (Fig. S5), arguing that electrophysiological defects in primary neurons are specific to NFT.

### Active electrical properties are altered in Tau neurons and restored by AdipoRon

**Fig. 5:**
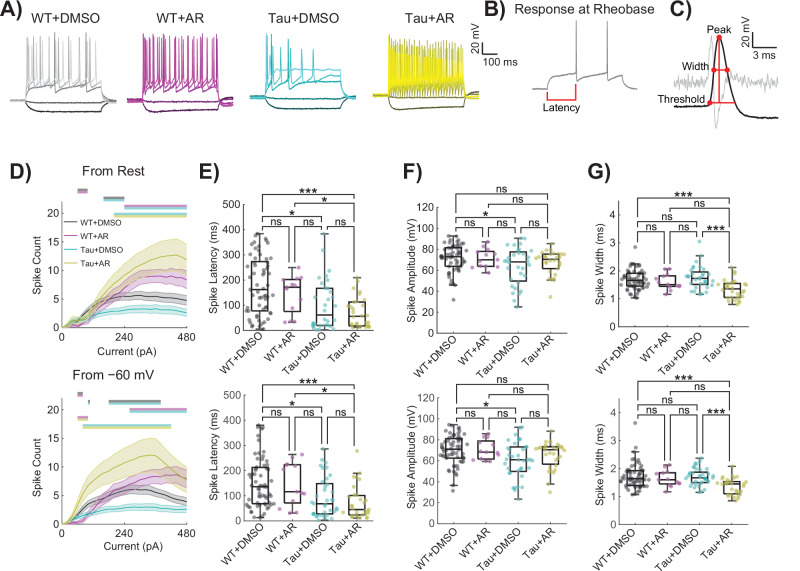
Active electrical properties are altered in Tau neurons and restored by AdipoRon. **A** Representative neuronal spiking patterns following 24-h 10 µM AR treatment using current applications of −100, −20, 160, 320, and 480 mV (darker to lighter shades). **B** Schematic voltage response at rheobase for a WT neuron, and **C** schematic of the first spike fired at rheobase with second derivative in light gray, computed rheobase parameters displayed by red dots and lines. **D** Action potential spike counts in response to 30 stepwise current applications of 20pA ranging −100 to 480pA, from rest (top) and from −60 mV (bottom). Repeated measures two-way ANOVA with Geisser–Greenhouse correction and post-hoc Tukey tests. Significant differences are shown as color-coded bars, mean ± SEM. Current–clamp Rheobase spike parameters from rest (top) and from −60 mV (bottom), including (**E**) spike latency, (**F**) amplitude, and (**G**) width for the first spike fired at rheobase, median ± IQR. Kruskal–Wallis with post-hoc Dunn’s tests. WT: 64 neurons, WT + AR: 13 neurons, Tau: 40 neurons, Tau+AR: 31 neurons. *p < 0.05, **p < 0.01, ***p < 0.001, ****p < 0.0001.

Active electrical properties involve voltage-gated ion channels that produce action potentials (i.e., spikes) that are widely considered the currency of “information” in the brain^71^. Given that disorders such as AD alter cognitive function, we compared spiking behavior between different AD models and WT (Fig. 5A). Several properties of spikes differed between Tau neurons and WT (Fig. 5B, C), and many of those properties were returned to near WT values by AR. Differences among WT and Tau neurons with respect to AR treatment were further investigated across stepwise current applications (Fig. 5D). A significant effect of genotype, treatment, and current step was detected in spike count as measured starting from the resting potential (Fig. 5D–G, top row). Since Tau neurons rested relatively depolarized compared to WT (Fig. 4I), a “standardized” spike count was also measured by holding near −60 mV (Fig. 5D–G, bottom row). Tukey multiple comparisons revealed that Tau neurons had reduced spike counts compared to all other groups, both from resting potential and −60 mV (Fig. 5D). Notably, Tau neurons sitting at their native resting potential were less likely to fire one or more spikes at higher current step applications, and from their resting potential and from −60 mV, tended to fire only a single spike unlike the WT or AR treated neurons (Fig. S6). AR-treated neurons of both genotypes were less likely to fire only a single action potential than untreated WT neurons for both recording conditions (Fig. S6).

At both resting potential and from a holding potential of −60 mV, Tau neurons firing action potentials at rheobase, the minimum current application necessary to elicit an action potential, were quicker to fire a spike from the onset of the current application (Fig. 5E). A short spike latency persisted for Tau neurons after AR treatment. Furthermore, Tau neurons had a reduced spike amplitude at the rheobase compared to WT neurons (Fig. 5F). Following AR treatment, Tau neurons fired spikes that were no longer significantly different in amplitude from WT neurons (Fig. 5F). Finally, Tau neurons with AR treatment had a reduced spike width compared to WT or Tau neurons without AR treatment (Fig. 5G). There were no statistical differences in the spike count across current steps from rest between WT and APP/PS1 neurons (Fig. S6). The results were similar for spike count across current steps from holding at −60 mV, nor were there differences at native resting potential in their tendency to fire one or more spikes across current steps (Fig. S6). These data reveal pervasive disruption in the electrical properties of neurons specific to NFT and provide evidence that at least some of the functional defects associated with Tau pathology can be corrected by treatment with AR. Indeed, the concentration of AR used here promotes very robust spiking. Further careful titration will be necessary to quantitatively mimic normal spiking properties in Tau neurons, especially in vivo or in the clinic.

### AdipoRon stimulates the activity of voltage-gated channels in Tau neurons

**Fig. 6:**
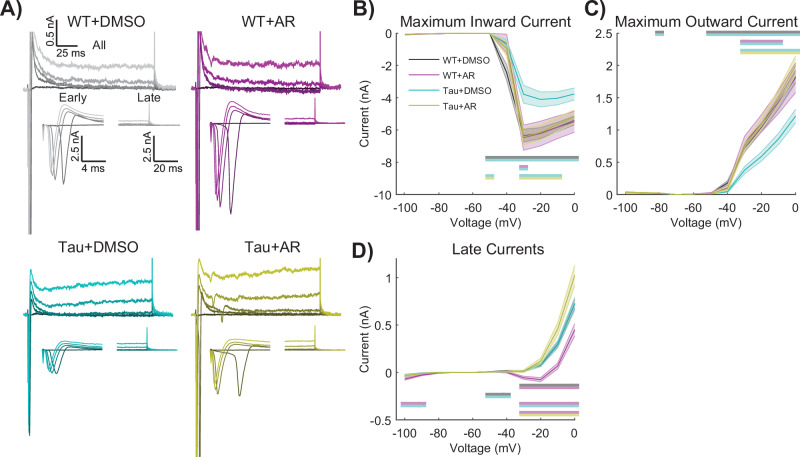
AdipoRon stimulates the activity of voltage-gated channels in Tau neurons. **A** Representative current responses of Tau and WT neurons following 24-treatment with 10 µM AR (or DMSO) in response to voltages of −40, −30, −20, −10, and 0 mV (darker to lighter shades). Stepwise voltage injections of 10 mV from 0–100 mV showing (**B**) maximum inward current, (**C**) maximum outward current, and (**D**) current–voltage curves. Repeated measures two-way ANOVA with Geisser–Greenhouse correction and post-hoc Tukey tests. Significant differences are shown as color-coded bars, mean ± SEM WT: 64 neurons, WT + AR: 13 neurons, Tau: 40 neurons, Tau+AR: 31 neurons.

The differences in spiking associated with Tau NFT and their rescue by AdipoRon prompted an investigation of voltage-gated ion channel activity. The activity of such channels can be quantified by controlling the neuronal membrane potential (i.e., voltage–clamp) and measuring the currents induced at different voltages and times. Although we are aware that highly arborized neurons cannot be accurately voltage–clamped (i.e., space–clamped) with current methods, we nevertheless found informative and statistically significant differences between genotypes and treatments (Fig. 6).

Using a range of applied voltages from a holding potential of −60 mV (Fig. 6A), the maximum inward current (i.e., likely an unclamped Na+ current) was measured for untreated and AR-treated WT and Tau neurons (Fig.6B). Tukey multiple comparisons revealed that Tau neurons had a reduced maximum inward current compared to all other groups. Significant main effects of genotype and treatment were detected, where AR had no effect on WT neurons but restored activity in Tau neurons to that of WT (Fig. 6B, Table S2). Maximum outward current (i.e., probably a fast, transient potassium current) of Tau neurons was less than all other groups, with significant main effects of genotype and treatment (Fig. 6C). AR had no impact on the maximum outward current in WT neurons but restored activity in Tau neurons to that of WT.

The maximum inward and outward currents occur within the first few milliseconds (i.e., “Early” in Fig. 6A). However, smaller, slower yet persistent currents (i.e., “Late” in Fig. 6A) are also likely to be important. These persistent currents were not different between WT and Tau neurons; however, the response to AR was genotype-specific (Fig. 6D). Multiple comparisons testing revealed that WT neurons with AR treatment had significantly larger net inward “Late” currents compared to all other groups (Fig. 6D). Identical voltage-clamp experiments showed no significant differences between WT and APP/PS1 neurons (Fig. S7). Together, the differences in inward/outward currents suggest that voltage-gated channel activity is compromised in Tau neurons and that AR treatment tends to favor greater intrinsic excitability, as both inward and outward voltage-gated currents are required for normal neuronal excitability.

## Discussion

Data presented here show the neuroprotective and restorative effects of the nonselective adiponectin receptor agonist AR in NFT-burdened primary cortical neurons. Mechanistically, this effect was shown to be AMPK-dependent and linked to the actions of Tau kinase GSK3b. Additional mechanisms for NFT clearance induced by AR include lysosomal genes, an observation that was corroborated in vitro by increases in puncta-associated p62 cargo protein and LC3b, a major component of mature autophagosome formation. Changes in autophagosomes were also dependent on AMPK. Energetic defects linked to NFT were restored by AR, including mitochondrial membrane potential and ATP availability. Neuronal functions such as synaptic communication, axonal transport, vesicle synthesis, dynamic arborization, and maintenance of electrophysiological capacity create energetic and anabolic demands. Electrophysiological defects in tauopathy mice have been previously reported^72^ and are consistent with the neuronal excitability defects reported here. It is unclear if the reduced availability of ATP is a major driver in NFT-associated loss of neuronal function or if other metabolic outcomes also play a role. For example, this study shows that Tau neurons display aberrant lipid accumulation. The greater lipid burden may be linked to inefficient or ineffective lipid fuel use. The positive enrichment of fatty acid metabolism genes with AR treatment is consistent with this idea. Another possibility is that blunted lipid mobilization impacts membrane-related cellular functions. Prior studies have linked lipid remodeling to adiponectin receptor signaling in kidney cells^73^, raising the possibility that the consequences of lipid accumulation in Tau neurons are not limited to problems of energy derivation.

This study shows reduced dendritic complexity in Tau neurons. The rescue of this phenotype by AR was JNK-dependent and AMPK-dependent. Although not tested in this study, it will be interesting to see if changes in arborization impact inter-cellular communications. Given the surprising lack of differences between WT and Tau neurons at the gene transcription level, it is unclear if synaptic processes, including assembly and presentation, are compromised by NFT. AR downregulated genes involved in synaptic transmission, an adjustment that, together with the increase in dendritic complexity, would be predicted to influence cell-cell communication. In primary hippocampal neurons, arborization and synaptic density are reliant on sufficiency in ATP production^74^. Electrophysiological properties are also heavily influenced by ATP availability. Resting potential depends mainly on the potassium gradient, set up by the ATP-dependent Na^+^/K^+^-ATPase, and on the resting K+ conductance, set up by the number and function of K^+^ channels. Voltage clamp experiments revealed that Tau neurons had reduced maximum inward and outward currents, but following 24-h AR treatment, they had maximum inward and outward currents that matched WT. Differences in voltage-gated channel expression were not detected at the mRNA level, indicating that functional differences could arise from changes at the protein or posttranslational level^75^. Impaired K+ channel function would contribute to higher input resistance and less healthy resting potential, whereas impaired Na+ channel function would contribute to defects in active properties (spiking), all of which were observed for Tau neurons. Tau neurons were less capable of generating action potentials and had altered spike properties. AR moved several of these spike properties to align with WT neurons. Ion channel production, localization, and function are all energy-dependent processes, indicating that rescue may be occurring via the improvement of cell metabolic health and ATP availability. The neuroactive ligand-receptor interaction pathway was the most enriched AR-responsive pathway and was negatively regulated in both WT and Tau neurons. Adiponectin is largely thought to modulate cellular metabolism, so this finding was unexpected. The functional significance of this adaptation is not clear but may be linked to changes in action potential firing and the avoidance of excitotoxicity caused by increased neurotransmitter release.

In contrast to the Tau neurons, we observed little impact of APP/PS1 in neuronal passive or active intrinsic excitability; however, this lack of difference in intrinsic electrical properties does not preclude synaptic communication along neuronal networks being disrupted by extracellular plaques. For Tau neurons, the electrophysiological defects reported here are coincident with defects in metabolism, proteostasis, soma size, and dendritic architecture. The intersectionality of these cellular phenotypes is not known. The AR-directed clearance of NFT is associated with mitochondrial rescue, increased available ATP, and activation of proteostasis. One possibility is that AR activates a regulatory hub that encompasses each of these phenotypes. Along these lines, studies in non-neuronal cells show that small changes in mitochondrial function have a broad impact on cellular processes related to fuel preference, energy storage, growth, and homeostasis^37^. Perhaps changes in mitochondrial function that occur within 2 h are the trigger to induce changes in other cellular processes, or maybe other aspects of intracellular signaling induced by AR create the framework for a coordinated response.

Several recent papers indicate that AR is effective in ameliorating the impact of amyloid pathology in AD models in vivo^76^ and that loss of adiponectin exacerbates amyloid pathology in wildtype and AD mouse models^77–79^. AR has been associated with enhanced insulin signaling in amyloid mouse models^77,80,81^. In older human patients with AD, elevated adiponectin has been reported to be positively correlated with CSF amyloid levels^82^, and the “adiponectin paradox” describes elevated adiponectin in end-stage diseases, including diabetes, cardiovascular disease, and AD^83–86^. It is unclear whether the increase in circulating adiponectin is a driver of morbidity or a consequence of a failed feedback loop due to loss of adiponectin sensitivity. This paradox has only been described in humans, and to date, it is unclear if it will be found in model organisms. It is also unclear whether problems with receptor activation by the endogenous ligand as a function of chronic disease would extend to a pharmacological strategy.

Compared to amyloid models of AD, fewer studies document the impact of AR on tauopathy, but a beneficial effect has been reported^29^. The data shown here, although promising, are limited to primary cell culture. In reality, neurons operate in an environment of diverse cell types, each with the ability to support and influence neuronal function. It will be important to extend the work to in vivo models, where neuronal function is assessed in situ and as part of a community of cells. Another limitation is that our study did not include an analysis of Tau isoform expression. Alternate splicing produces different isoforms in humans that differ structurally and regionally in their accumulation in a disease-specific manner^87^, but none of that biology is interrogated in this study. Another consideration is that the use of the transgenic seeded neurons reflects the condition of tauopathy but cannot inform about the etiology of the condition. Finally, the treatment of primary Tau neurons with AR, having been inspired by studies of metabolic regulation in the context of caloric restriction, has demonstrated a broader beneficial effect than had been anticipated. Distinct cellular pathways are linked to adiponectin signaling here and involve critical kinases AMPK, GSK3b, and JNK, but the specific mechanistic connections and order in which they are recruited have not been established. Taken together, our data show that NFTs impinge on multiple aspects of neuronal health and function, and features such as mitochondrial metabolism, autophagy, and neuronal excitability can be harnessed in a coordinated manner through adiponectin receptor activation. Should our findings stand up in vivo, then a strategy to target adiponectin signaling could be relevant for the treatment of NFT. The stage is set for preclinical studies to determine the validity of this promising agent in mouse models of tauopathy and to confirm the conservation of efficacy as a function of age and disease progression.

## Methods

### Cell culture

Primary cortical neurons were isolated from the brains of P0 hTau P301S and APP/PS1 mice. Briefly, the cortices were harvested from pups into ice-cold HBSS, where the midbrain and meninges were removed. Brain tissue was minced and digested in 0.25% trypsin for 20 min at 37 °C. Trypsin was quenched with DMEM/10%FBS/1%Pen/Strep, and cells were dissociated and counted prior to plating on poly-d-lysine coated plates. Cultures were then fully changed to Neurobasal Plus Media (2% B27^+^, 1% GlutaMax, 1% Pen/Strep) the subsequent day. Upon the first change to Neurobasal media, neurons were treated with 1 μM AraC (cytosine arabinoside) to eliminate glial cell populations. Media was changed by ½ volume every 3–4 days until experiments. To induce neurofibrillary tau tangle accumulation in the P301S, transgenic neurons were seeded with 3 μg/mL of pre-formed fibrils (PFF) (StressMarq) on day 7 in vitro and then cultured for 18 days before analysis^23^. All primary neuron experiments were conducted between days 25–38 in vitro. APP/PS1 neuron cultures showed elevated levels of Aβ and, therefore, were not seeded with exogenous protein. For electrophysiology experiments, cells were cultured as described, except glial cells were maintained in culture by excluding AraC from the initial Neurobasal Plus Media addition. Because these are primary neurons and not immortalized cell lines, we employed a strategy where each cell was considered individually for data capture and subsequent analysis. For all experiments apart from the RNASeq, no less than two independent sessions were conducted using littermates within each session.

#### Drug treatments

Cells were treated with 10 μM AdipoRon or DMSO (vehicle control) prior to all experiments, as shown above. Treatment was accompanied by a 50% media change. For a 10-minute treatment, AdipoRon was added as a 5× solution diluted to a final 1× in the culture media. Compound C (Millipore Sigma; 171260) treatment was co-administered with AR during 50% media change as an 8 μM dose. SP600125 (MedChem Express; HY-12041) was co-administered with AR during 50% media change as a 10 μM dose. BAY-3827 was administered at a 1 μM dose. Lithium chloride (Sigma Aldrich; L7026) was administered for 24 h through a 50% media change at a final concentration of 15 mM.

### Immunofluorescence

Each imaging data capture experiment involved at least 5–7 individual cells per animal per treatment per session, with data reported from a minimum of two independent sessions. Immunofluorescence detection was conducted using standard techniques, and images were acquired using a Leica DMi8 inverted microscope equipped with an HCX PL Fluotar 100×/1.30 oil objective. Images were analyzed using ImageJ (NIH, Wayne Rasband, http://rsb.info.nih.gov/ij/). Cells were grown on glass coverslips coated with poly-d-lysine. Cells were fixed in 10% formalin for 10 min, permeabilized with 0.3% Triton X-100 in PBS for 1 h, and incubated with a primary antibody cocktail overnight. Primary antibodies used: MAP2 (ab5392; Abcam), AT-8 (MN1020; Thermo Scientific), MC-1 (Peter Davies lab), LC3b (ab48394; Abcam), p62 (23214; Cell Signaling Technologies), pGSK3b (Ser9) (9336; Cell Signaling Technologies), GSK3b (9832; Cell Signaling Technologies, pAMPK (Thr172) (2535; Cell Signaling Technologies), AMPK (2532; Cell Signaling Technologies), Tomm20 (ab56783; Abcam), pJNK (Thr183/ Tyr185 (9255; Cell Signaling Technologies), and JNK (9252; Cell Signaling Technologies). Lipid droplets were detected using HCS LipidTox Red following the manufacturer’s protocol (H34476; Thermo Scientific). Sholl analysis was conducted using the SNT plugin (https://imagej.net/plugins/snt/) for ImageJ.

### Western blotting

Cells were lysed, and protein was extracted in a modified RIPA buffer containing protease and phosphatase inhibitors (P8340 and 524624, respectively; Sigma-Aldrich, St. Louis, MO, USA). Proteins were detected by immunoblotting using standard techniques. Western blot densitometry was conducted in ImageJ to detect band intensity. All proteins were normalized to total protein in the lane detected by the Ponceau stain. Antibodies used were LC3b (ab48394; Abcam), LAMP2A (51-2200; Thermo Scientific), pTau AT-8 (MN1020; Thermo Scientific), and total Tau (10274-1-AP; Proteintech).

### RNA sequencing

RNA extraction was completed using a Direct-zol RNA kit (Zymo Research) according to the manufacturer’s instructions. Each RNA library was generated following the Illumina TruSeq RNA Sample Preparation Guide and the Illumina TruSeq RNA Sample Preparation. Purified total RNA was used to generate mRNA libraries using NEBNext Poly(A) mRNA Magnetic Isolation Module and NEBNext Ultra RNA Library Prep kit for Illumina (Illumina Inc.). Quality and quantity were assessed using an Agilent DNA1000 series chip assay and Invitrogen Qubit HS Kit (Invitrogen), respectively. Sequencing reads were trimmed to remove sequencing adapters and low-quality bases, aligned to the mm10 reference genome using the STAR aligner, and alignments used as input to RSEM for quantification. Differential gene expression analysis was performed via EdgeR generalized linear model (GLM) method.

#### String analysis

Markov Clustering Algorithm (MCL) was used with an inflation parameter of 3. Active interaction sources used: Experiments, databases, co-expression, neighborhood, gene fusion, co-occurrence. The minimum required interaction score was 0.700.

### ATP luminescence assay

Relative ATP levels were quantified with the ATPlite assay (Perkin-Elmer). Luminescence was measured using an Infinite M200 microplate reader (TECAN). Neurons were cultured as above and treated with vehicle or AdipoRon (10 μM) for 12 h.

### JC-1 assay

Mitochondrial membrane potential was quantified with JC-1 dye (Invitrogen). Neurons were treated for 2 h and then incubated with 1 µg/mL JC-1 dye for 30 min. Cells were washed with PBS prior to fluorescence detection. Fluorescence was measured using excitation/emission wavelengths of 535/590 nm and 485/530 nm.

### qRT-PCR

Cells were treated as outlined above and RNA was collected 24 h after treatment. For NSC differentiation and primary neuron maturation, cells were lysed at day in vitro indicated above. Cells were lysed with Trizol and RNA was isolated using the Zymo Research Direct-zol RNA MiniPrep Kit. RT-qPCR was conducted using iTaq Universal SYBR Green Supermix (1725121, Bio-Rad).

### In vitro electrophysiology recordings

Recordings were performed on cultured neurons described above in Cell Culture. All recordings were done in an extracellular solution containing (in mM) 145 NaCl, 2.5 KCl, 1 MgCl_2_, 2 CaCl_2_, 10 HEPES, and 10 dextrose. The extracellular solution was adjusted to a pH of 7.3 with 5 N NaOH and 320–325 mOsm with sucrose. Whole-cell patch-clamp recordings were made using an upright microscope (Axioskop FS2, Zeiss) with infrared differential interference contrast optics. Patch pipettes pulled from thin-walled borosilicate glass (World Precision Instruments) had a resistance of 3–5 MΩ when filled with intracellular solution containing (in mM): 135 K-gluconate, 5 KCl, 0.1 EGTA, 10 HEPES, 2 MgATP, 0.3 Na_2_GTP, 0.25 CaCl_2_, and 20 Na_2_-phosophocreatine. The intracellular solution was adjusted to a pH of 7.2 with 5 N KOH and 310–315 mOsm with H_2_O. Recordings were done using an Axopatch 200B or MultiClamp 700B amplifier (Axon Instruments), filtered at 5 kHz using a 4-pole Bessel filter, and digitized at 10 kHz using a Digidata 1320 A or 1322 A analog-digital interface (Axon Instruments). Data were acquired to a Power Mac G4 (Apple) using Axograph X v1.5.4 (Axograph.com). Input resistance, series resistance, and capacitance were assessed from the fit of the current responses to −5 mV pulses while holding the potential at −60 mV. Three protocols were run on each cell: a voltage-clamp and two current–clamps, one run from the cell’s resting potential and another run from holding the cell at −60 mV. The voltage–clamp protocol was performed from holding cells at −60 mV. The current clamp protocol consisted of 20 pA current steps spaced between −100 and 480 pA, repeated five times. Neuronal recordings that were used for analysis were stable across the entire recording session, as measured by monitoring consistent series resistance and resting potential. All spike analysis was performed in MATLAB vR2022b with custom scripts. Scripts are publicly available at https://github.com/DannyLasky/PatchClamp.

#### Electrophysiology data analysis

Resistance, capacitance, and resting potential were assessed initially and following each of the protocols. Spikes were detected by an upward slope that crossed 30 mV/ms, followed in the next 10 ms by a downward slope that crossed −15 mV/ms. Each spike was additionally required to pass above 0 mV. Spontaneous and rebound spikes were omitted from the analysis, which were collectively defined as spikes occurring outside of a depolarizing current application window. Additional computations were performed when analyzing the first spike fired at rheobase, which is defined as the minimum current application necessary to elicit a spike. The maximum in the second derivative was found within a window spanning from the 30 mV/ms up the cross, used for our spike detection, to 3 ms prior. This effectively located the greatest curvature of the spike, a marker of spike initiation. We defined the voltage of the original signal at this time point as “threshold,” the voltage at which the cell fired action potentials. The end of the spike was defined as when the spike crossed back under the threshold. Latency was defined as the time from the beginning of the current application to the spike threshold. We computed spike amplitude as the voltage from the threshold to the peak of the spike. Finally, we defined spike width as the full width at half amplitude. On rare occasions (<1% of data), a first spike fired at rheobase did not cross back under its threshold. In this case, width could not be computed, so the spike was excluded from the additional calculations.

Voltage-clamp traces were leak subtracted. The smallest negative step (−10 mV) was used as the best measure of passive current flowing through leak channels and capacitance. The current at this voltage step was scaled in a linear ohmic manner dependent on the voltage applied and then subtracted from the original signal. Following leak subtraction, the traces were zeroed using the current prior to applying a voltage step. Maximum inward and outward currents were computed from the first 10 ms following voltage application. Steady-state currents for the IV curves were computed from the average current in the last third of the voltage application. Importantly, these voltage-clamp data were not space-clamped and did not properly account for the arborization of neurons. No liquid junction offsets were applied.

### Statistics

All data capture experiments were conducted in samples derived from a minimum of four pups per genotype unless otherwise noted. For all experiments apart from the RNASeq, at least two independent sessions were conducted using littermates within each session. Each image-based data capture experiment involved at least five individual cells per animal per treatment per session. Because these are primary neurons and not immortalized cell lines, we employed a strategy where each cell was considered individually for data capture and subsequent analysis. Outliers were identified by ROUT using a threshold of *Q* = 0.05. Typically, datasets involving two groups were analyzed by unpaired Student’s *t*-test and for datasets with three groups, Brown–Forsythe and Welch one-way ANOVA was used. For datasets with four groups, two-way ANOVA with Tukey’s Multiple comparisons test was used. Sholl analyses were statistically assessed by a two-way ANOVA of the area under the curves. Data for intrinsic excitability (Fig. 4, Fig. S5) and rheobase parameters (Fig. 5, Fig. S6) were non-normal and analyzed appropriately with Kruskal–Wallis and post-hoc Dunn’s tests for the Tau data and Mann–Whitney *U* tests for the APP/PS1 data. Data for spike count across the current step, maximum inward and outward currents, and IV curves were analyzed with repeated measures of two-way ANOVA with Geisser–Greenhouse correction^88^ and post-hoc Tukey tests. One or more spikes and exactly one spike data were analyzed via *F*-test, fitting one-phase association curves through the origin to one plus spike plots and fitting lines through the origin to exactly one spike plot. The equation for these curves is Y = Plateau * (1 − exp(−K*x)). For all statistics, *p* < 0.05 was defined as significant. Asterisks on figures denote significance levels: *p < 0.05, **p < 0.01, ***p < 0.001, ****p < 0.0001.

### Reporting summary

Further information on research design is available in the Nature Portfolio Reporting Summary linked to this article.

## Data accessibility

All RNA sequencing data from this study are deposited in the GEO repository (GSE255813). The source data underlying all figures, including uncropped images of immunoblots, can be found in the supplemental information (Fig. S8). All other datasets are available from the corresponding author upon reasonable request.

## Supplementary information

Supplementary Information

Table S1

Description of Additional Supplementary Files

Supplementary Data

Reporting summary
