# Supplementary material for: Reversal of neuronal tau pathology via adiponectin receptor activation
Source: Commun Biol. 2025 Jan 4;8:8. doi: 10.1038/s42003-024-07391-z (PMC11700159; doi:10.1038/s42003-024-07391-z)
Supplement: Supplementary file 1 — Supplementary Information [file 42003_2024_7391_MOESM1_ESM.pdf]

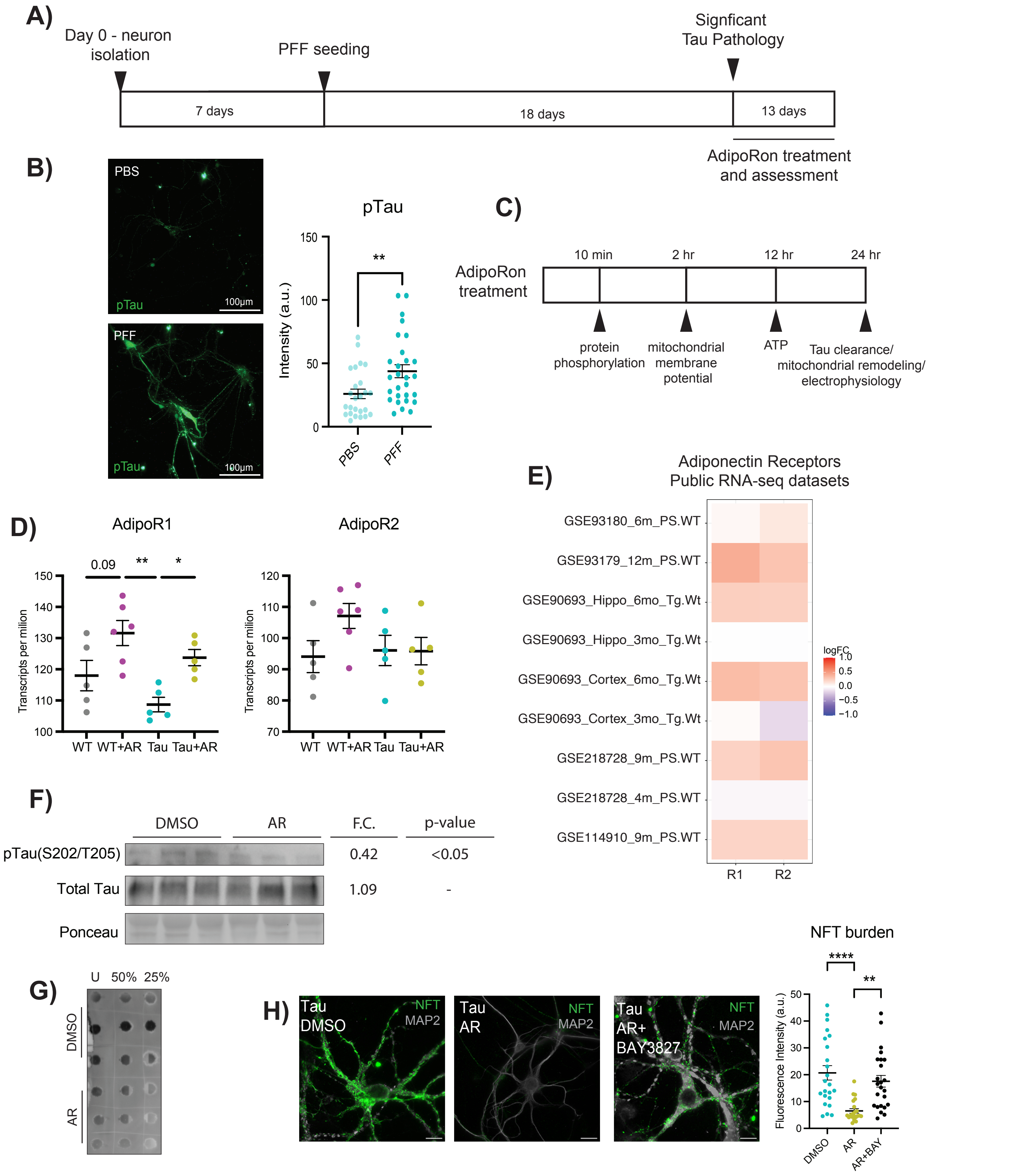

Figure S1 (related to Fig.1): A. Schematic of neuron culture timeline. B. Immunofluorescence of phosphorylated tau (AT-8) in Tau neurons seeded with PFF or PBS control (n = 3 mice per treatment, n=25–28 neurons per treatment) scale bar 100µm. C. Schematic of AdipoRon treatment times used in this paper. D. Adiponectin receptor expression from the RNA-seq dataset (n = 5-6 mice per treatment per genotype). E. Heatmap of adipoR1/2 expression in publicly available RNA-seq datasets. F. Western blot of pTau detected in DMSO or 10µM AR-treated Tau neurons (n=3 mice per treatment). G. Immunofluorescence of NFT in Tau neurons following 24-hour treatment with 10µM AR ± 1µM AMPK inhibitor BAY3827 (n = 3 mice per treatment, n = 22-24 neurons per treatment). Significance determined by Student's t-test with Welch's correction or two-way one-way ANOVA with Tukey's multiple comparisons test. Data shown as mean ± SEM.

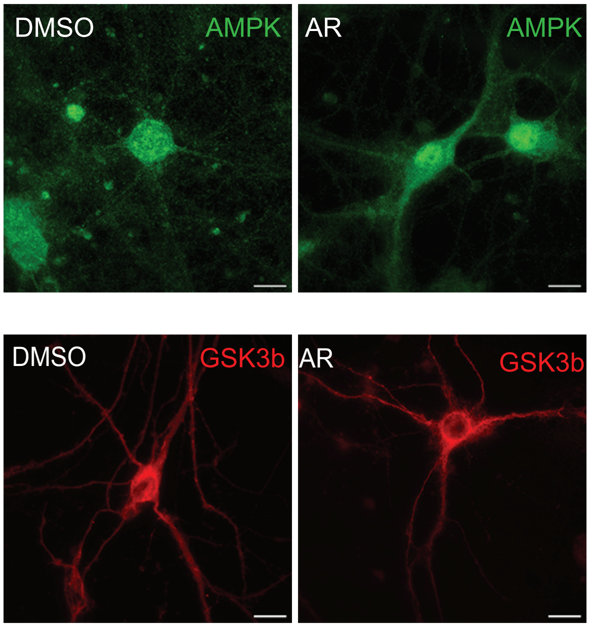

Figure S2 (related to Fig.1): Representative immunofluorescence images of total protein levels of AMPK and GSK3b. Quantification is shown in Fig 1.

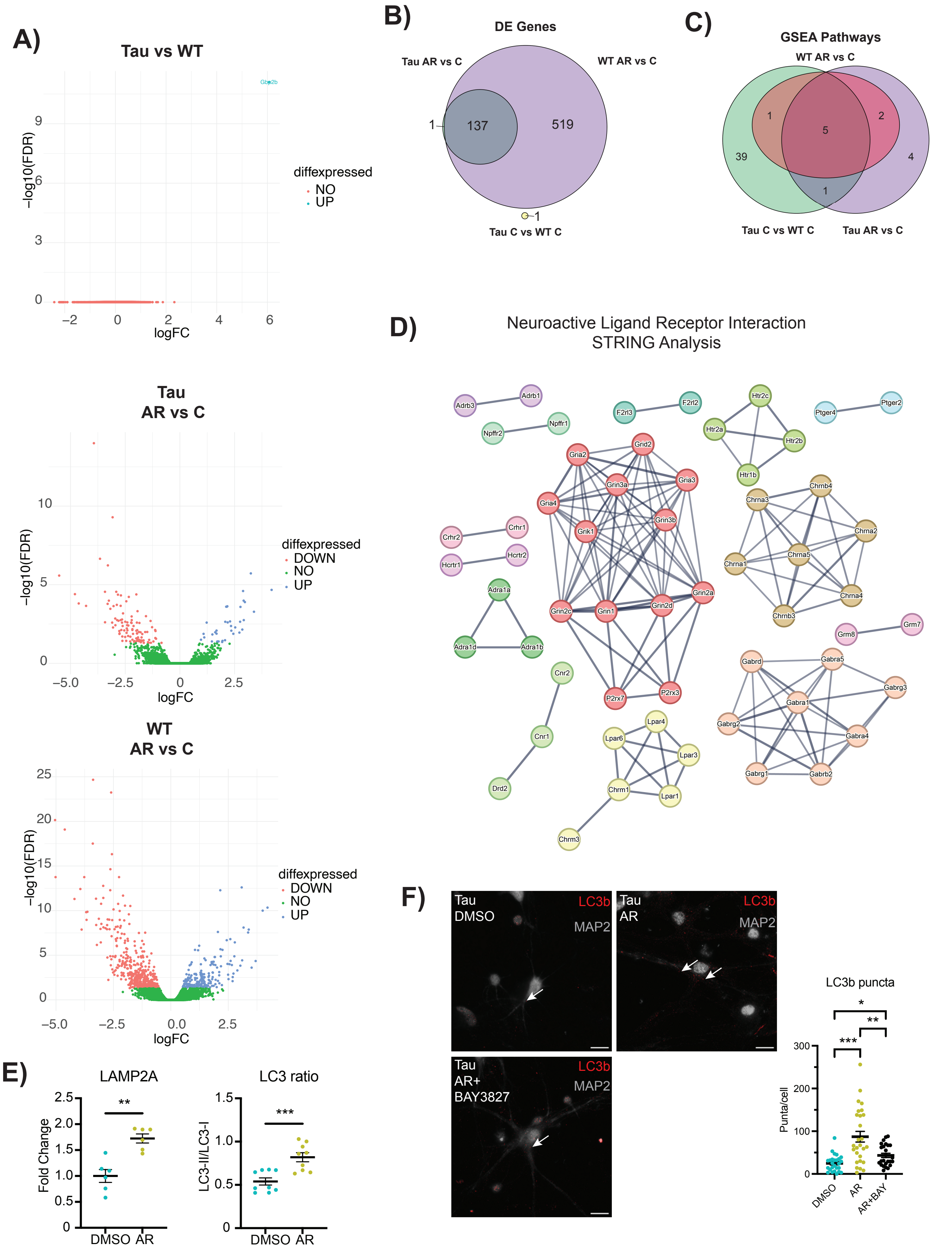

Figure S3 (related to Fig.2): A. Volcano plots displaying the differentially expressed genes in RNAseq data. B. Venn diagram showing overlap between differentially expressed genes in the three comparisons above. C. Venn diagram showing the overlap between enriched KEGG pathways in the three comparisons above. D. Complete STRING diagram from the Neuroactive Ligand Receptor interaction pathway. Related to Fig.2C. E. Quantification of western blots shown in 2G. F. Immunofluorescent detection and quantification of autophagosome marker LC3b in Tau neurons after 24-hour treatment with 10 μM AdipoRon ± 1μM BAY-3827 (n=3 mice per treatment, n=25-27 neurons per treatment).

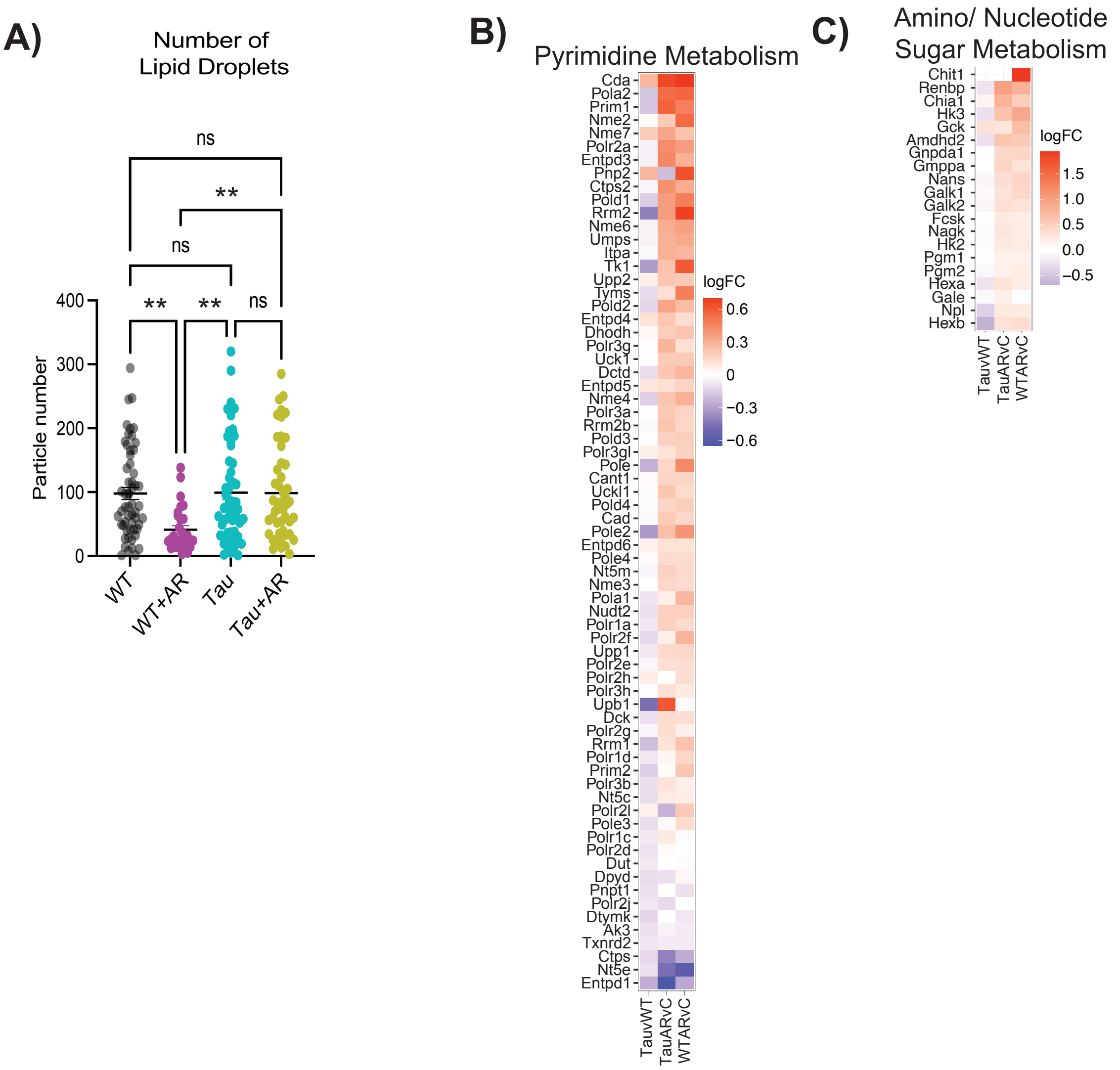

Figure S4 (related to Fig.3): A. Quantification of lipid droplet number from neurons shown in Fig.3I. Data shown as mean ± SEM. Heatmaps of B. pyrimidine metabolism and C. amino/ nucleotide sugar metabolism identified via GSEA pathways in AR-treated WT and Tau neurons.

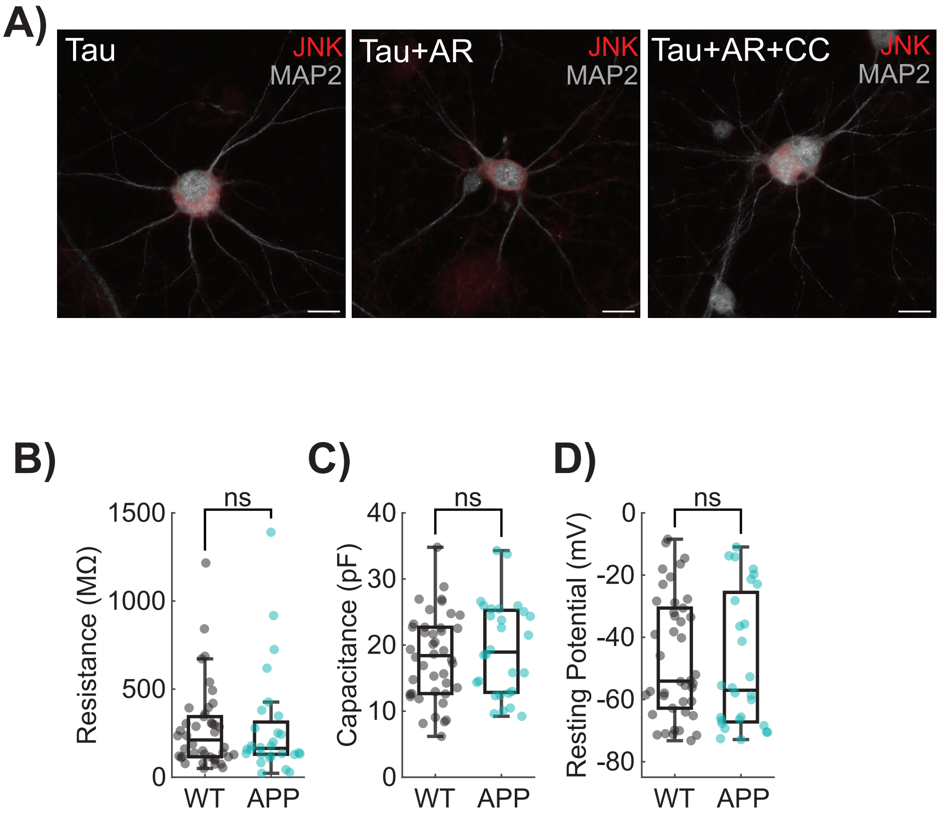

Figure S5 (related to Fig.4). A. Representative immunofluorescence images of total protein levels of JNK, with quantification shown in Fig.4D. Passive excitability including B. resistance, C. capacitance, and D. resting potential for APP/PS1 and WT neurons shown as median ± IQR. Mann–Whitney U tests. WT: 42 neurons, APP/PS1: 28 neurons.

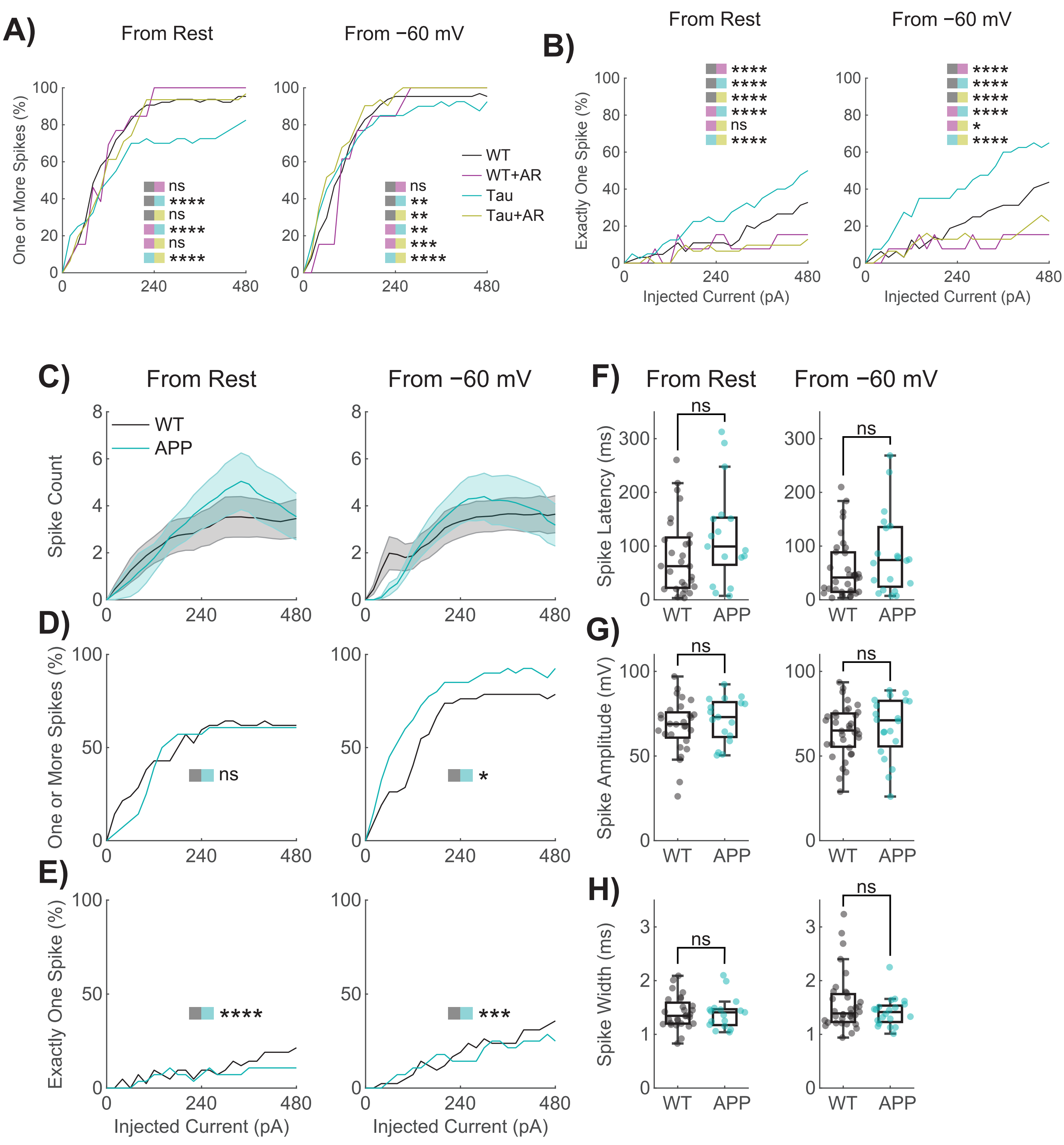

Figure S6 (related to Fig.5): A. The percentage of cells firing one or more spikes or B. exactly one spike. Significance between groups is displayed as paired colored blocks. Sample sizes are WT: 64 neurons, WT+AR: 13 neurons, Tau: 40 neurons, Tau+AR: 31 neurons. C. Action potential spike counts for APP/PS1 and WT neurons were measured in response to 30 stepwise current applications of 20pA from −100–480pA for each cell from rest (left) and from −60mV (right). Repeated measures two-way ANOVA with Geisser–Greenhouse correction and post-hoc Tukey tests. Mean ± SEM. D. The percentage of cells firing one or more spikes or E. exactly one spike was measured across current steps. Significance between groups is displayed as paired colored blocks. Sample sizes for C-E are WT: 42, APP/PS1: 28. Rheobase spike F. latency, G. amplitude, and H. width for the first spike fired at rheobase collected from current-clamp protocols performed from rest (left) and from −60mV (right) median ± IQR. Mann–Whitney U tests. Sample sizes for F-G are WT: 28 neurons, APP: 17 neurons from rest and WT: 35 neurons, APP/PS1: 20 neurons from −60 mV. * $p<0.05$, *** $p<0.001$, **** $p<0.0001$.

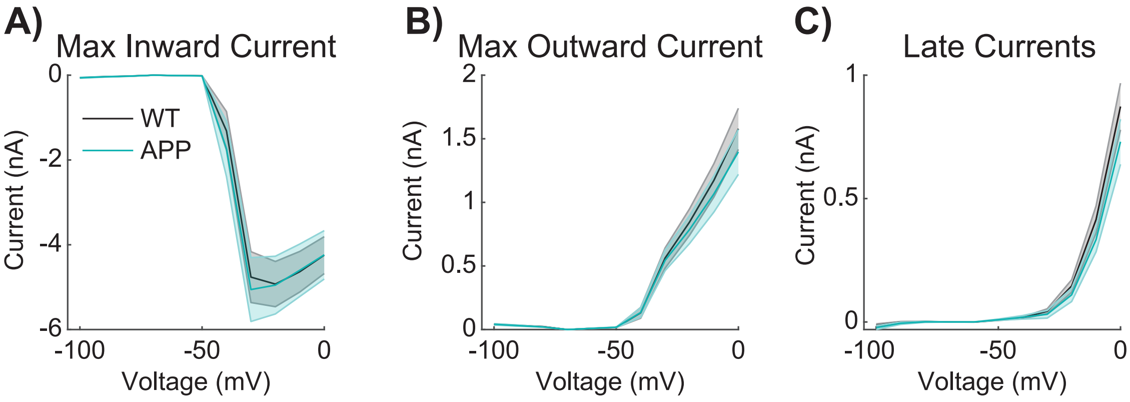

Figure S7 (related to Fig.6): Stepwise voltage injections of 10mV from 0–100mV showing A. the maximum inward current, B. outward current, and C. IV curve (C) in APP/PS1 and WT neurons. Statistics were performed using repeated measures two-way ANOVA with Geisser–Greenhouse correction and post-hoc Tukey tests. Error bars are SEM. Sample sizes are WT: 42 neurons and APP/PS1: 28 neurons.

Fig 2G - LAMP2A

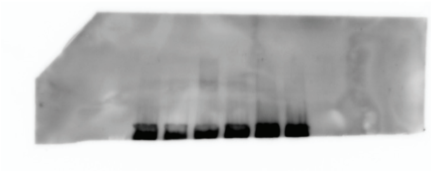

Fig 2G - LC3

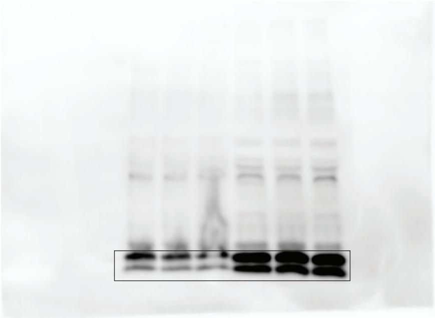

Fig S1F - pTau

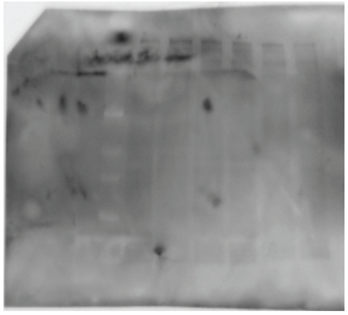

Fig S1F - Total Tau

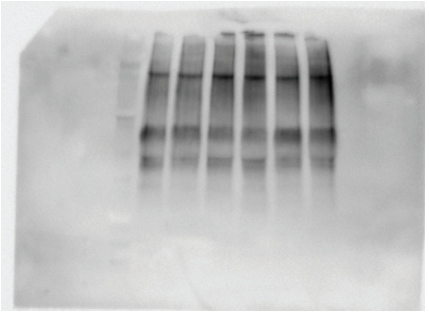

Figure S8. Raw western blot images for figures 2 and S1.

Table S1. Differentially expressed genes detected by RNA seq in WT and Tau neurons treated with AdipoRon.
- uploaded separately in an excel file.

Voltage clamp measurement	Main group effect	Main Voltage effect	Group x Voltage interaction effect
Maximum Inward Current	$F_{(3,144)}$=6.227, p=0.0005	$F_{(1.827,263.1)}$=298.8, p<0.0001	$F_{(30,1440)}$=4.709, p<0.0001
Maximum Outward Current	$F_{(3,144)}$=8.721, p<0.0001	$F_{(1.174,169)}$=450.3, p<0.0001	$F_{(30,1440)}$=7.820, p<0.0001
IV Curve	$F_{(3,144)}$ = 7.269, p=0.0001	$F_{(1.132,163)}$ = 258.2, p<0.0001	$F_{(30,1440)}$ = 6.175, p<0.0001

Table S2. AdipoRon changes voltage-gated channel activity in Tau neurons.
