# Supplementary material for: Reversal of neuronal tau pathology via adiponectin receptor activation
Source: Commun Biol. 2025 Jan 4;8:8. doi: 10.1038/s42003-024-07391-z (PMC11700159; doi:10.1038/s42003-024-07391-z)
Supplement: Supplementary file 3 — Description of Additional Supplementary Files [file 42003_2024_7391_MOESM3_ESM.pdf]

Description of Additional Supplementary Files

File name: Supplementary Data
Description: Source data for all figures and supplemental figures
